# Drug-associated congenital anomalies of the external ear identified in the United States food and drug administration adverse event reporting system database

**DOI:** 10.1038/s41598-024-74744-3

**Published:** 2024-10-09

**Authors:** Xinyu Li, Jiajia Hao, Datao Li, Ruhong Zhang

**Affiliations:** Department of Plastic and Reconstructive Surgery, Shanghai Ninth People’s Hospital, Shanghai Jiao Tong University School of Medicine, Shanghai, China

**Keywords:** Congenital anomalies, FAERS, Disproportionality analysis, Pharmacovigilance, Drug discovery, Drug safety, Drug screening

## Abstract

Congenital anomalies of the external ear can have a significant impact on a child’s development and quality of life. While genetic factors play a crucial role in the etiology of these anomalies, environmental factors such as drug exposure during pregnancy may also contribute to their occurrence. This study aims to investigate the association between drug exposure and congenital anomalies of the external ear using data from an adverse drug reaction report database. Using OpenVigil 2.1, we queried the FAERS database to retrieve adverse event reports from the first quarter of 2004 to the first quarter of 2024. To identify relevant cases, we used Medical Dictionary for Regulatory Activities terms focusing on congenital anomalies of the external ear. Drug generic names were sourced from the DrugBank database. To assess safety signals and rank drugs by their signal strength, we conducted a disproportionality analysis, generating reporting odds ratios (ROR) and proportional reporting ratios (PRR). A total of 20,754,281 AE reports were identified in the FAERS database from Q1 2004 to Q1 2024, of which 1763 were related to congenital anomalies of the external ear. Valproic acid (122 cases) was associated with the most cases, followed by mycophenolate mofetil (105 cases) and lamotrigine (65 cases). According to the disproportionality analysis, the top five drugs with the highest ROR and PRR were primidone (ROR: 397.05, 95% CI 147.21, 1070.9; PRR: 388.71, 95% CI 145.89, 1035.7), valproic acid (ROR: 239.46, 95% CI 123.75, 463.37; PRR: 236.42, 95% CI 123.82, 451.43), tapazole (ROR: 198.35, 95% CI 63.49, 619.67; PRR: 196.25, 95% CI 62.97, 611.67), nevirapine (ROR: 138.24, 95% CI 82.9, 230.51; PRR: 137.23, 95% CI 82.44, 228.44), and sebivo (ROR: 117.1, 95% CI 48.51, 282.67; PRR: 116.37, 95% CI 48.17, 281.12). This study identified several drugs significantly associated with congenital anomalies of the external ear in the FAERS database using disproportionality analysis. The findings can help healthcare professionals better recognize and manage drug-induced congenital anomalies of the external ear, particularly when prescribing high-risk medications. Further research is needed to elucidate the mechanisms underlying these associations and develop strategies for preventing and mitigating drug-induced congenital anomalies of the external ear.

## Introduction

Congenital anomalies of the external ear, also known as microtia or anotia, are rare birth defects that can significantly impact an individual’s appearance, hearing, and overall quality of life^1,2^. While the etiology of these anomalies is not fully understood, genetic factors and environmental exposures, including certain medications, have been implicated as potential risk factors^3–5^. Maternal drug use during pregnancy is a well-recognized risk factor for various birth defects, as these drugs can interfere with normal fetal development, particularly during the first trimester when organogenesis occurs. Previous studies have examined the link between various medications and the incidence of congenital ear malformations. However, the findings have been inconsistent and frequently constrained by small sample sizes, retrospective data, self-reported medication use, and methodological challenges^6–9^. For instance, a study found an increased risk of microtia associated with the use of pseudo ephedrine during pregnancy^10^, while another study did not find a significant association between the use of decongestants and the development of ear defects^11^. These conflicting results highlight the need for further research to clarify the potential role of specific drugs in the etiology of external ear anomalies. Furthermore, the mechanisms underlying the potential teratogenic effects of drugs on the developing ear remain poorly understood. Animal studies have provided some insights into the molecular pathways and cellular processes involved in ear development, but the extrapolation of these findings to humans is challenging due to species-specific differences in embryonic development and drug metabolism^12^.

Given the limited and conflicting evidence, there is a pressing need for more comprehensive and well-designed studies to address the unresolved research gaps and provide a clearer understanding of the association between medications and congenital anomalies of the external ear. The identification of specific drugs that may increase the risk of these birth defects is crucial for informing clinical decision-making, guiding prenatal counseling, and developing targeted prevention strategies. In this study, we aim to address these limitations by leveraging the United States Food and Drug Administration Adverse Event Reporting System (FAERS) database to identify potential drug-associated congenital anomalies of the external ear. By analyzing a large, national-level dataset, we seek to provide a more comprehensive assessment of the relationship between specific medications and the occurrence of these rare birth defects, while accounting for potential confounding factors. Our findings may contribute to a better understanding of the underlying mechanisms and inform future research efforts to elucidate the complex interplay between drugs and ear development.

## Methods

### Data source

This retrospective pharmacovigilance study utilized the FDA Adverse Event Reporting System (FAERS) database as its primary data source. FAERS (https://fis.fda.gov/extensions/FPD-QDE-FAERS/FPD-QDE-FAERS.html) is a critical tool for post-marketing surveillance and evaluation of drugs and biological products^13^. It is a publicly accessible repository containing individual case safety reports (ICSRs) of suspected adverse drug reactions, submitted by healthcare professionals, consumers, and pharmaceutical companies. Our study focused specifically on ICSRs reporting drug-induced congenital anomalies of the external ear. We extracted data from the FAERS quarterly data files, which contain raw data including demographic and administrative information, drug details, reaction information, patient outcomes, and reporting sources. Each ICSR was treated as a single adverse event (AE) report for analysis purposes. This study was exempt from institutional review board ethics approval due to the anonymously coded design of the FAERS database. It comprises extensive data on adverse events (AEs) and medication errors that have been reported to the FDA. These data are primarily sourced from healthcare professionals and consumers and encompass a broad range of information, including patient demographic details, specific drug information, and clinical outcomes associated with the use of these pharmaceuticals.

Our research employed the OpenVigil 2.1 pharmacovigilance analytics platform, accessible at http://h2876314.stratoserver.net:8080/OV2/search/, for querying the FAERS database^14^. OpenVigil 2.1, a publicly accessible tool in the field of pharmacovigilance, is adept at importing raw AE data from FAERS. It is designed to execute a range of functions including data extraction, cleaning, mining, and analysis, as cited in references. The platform, diligently updated and maintained by Böhm R and colleagues, currently hosts FAERS data extending from the first quarter of 2004 through to the first quarter of 2024. This extensive dataset comprises a total of 50,659,288 recorded adverse events.

We extracted adverse event reports from the FAERS database for the time frame from first quarter of 2004 through to the first quarter of 2024. In this study, the reports included severe adverse events, such as hospitalization, disability, or death. The types of reports were classified by FAERS as direct, expedited, or periodic. To reduce the impact of confounding factors, we chose the direct reporting type (submitted to FDA by consumers or healthcare professionals). The primary selection criterion focused on “primary suspect” drugs. “Secondary suspect” drugs were excluded due to the higher uncertainty regarding their association with the reported adverse events.

### Definition of AEs and drugs

AEs from the FAERS database were categorized using the Preferred Term (PT) in the Medical Dictionary for Regulatory Activities (MedDRA, Version 24.0). The PT hierarchy in MedDRA is structured at level 4, a commonly used level in such analyses^15^. To investigate anomaly of external ear congenital-related drug events, we searched for accessory auricle (MedDRA code: 10,000,361), anomaly of external ear congenital (MedDRA code: 10,062,339), anotia (MedDRA code: 10,002,654), congenital aural fistula (MedDRA code: c), constricted ear deformity (MedDRA code: 10,071,233), external auditory canal atresia (MedDRA code: 10,054,875), low set ears (MedDRA code: 10,024,929), macrotia (MedDRA code: 10,024,929), microtia (MedDRA code: 10,027,555), protuberant ear (MedDRA code: 10,071,232) in the PT column. Subsequently, all reports related to congenital anomalies of the external ear were downloaded. For statistical analysis, we used the generic name as the unique identifier for each drug. If the drug name used could not be retrieved in the DrugBank database (https://go.drugbank.com/drugs), we considered it an incorrect report and performed manual elimination.

### Statistical analysis

A descriptive analysis was performed to provide an overview of the clinical characteristics of patients suffering from drug-induced anomaly of external ear congenital. The analysis included various factors such as age, gender, indication, outcome, and the country from which the reports originated. Each individual safety report was treated as a single AE report for the purpose of this study. Disproportionality analysis was utilized to formulate hypotheses regarding potential associations between medications and anomaly of external ear congenital. This analytical approach involves calculating the ratio of observed to expected drug-adverse event pairs, which is deemed disproportionate if it surpasses a predetermined threshold^16^. The reporting odds ratio (ROR) and proportional reporting ratio (PRR) are the two most frequently employed frequency-based methods in disproportionality analysis^19^. These methods are popular due to their straightforward calculation and the consistent nature of their results^17–19^. While these measures are conceptually similar, each offers unique strengths in signal detection. The PRR is widely used in pharmacovigilance and provides a straightforward interpretation of the relative reporting rate. In contrast, the ROR is particularly useful when the adverse event of interest is rare, and it allows for adjustment of confounding factors in more complex analyses. Using both measures allows for cross-validation of signals, reducing the likelihood of false positives or negatives. The calculation equations and criteria were listed in Table 1. We defined a disproportionality signal as a case where the lower bound of the 95% confidence interval of the PRR was greater than 1, and the number of cases was at least 3. A comparatively elevated ROR or PRR suggests a more robust statistical association between the suspected medication and the adverse event under investigation. The OpenVigil 2.1 platform automatically computed the ROR and PRR values, and the resulting data were subsequently processed and analyzed using Microsoft Excel 2019 software. The study was exempt from institutional review board ethics approval due to the anonymously coded design of the FAERS database.

**Table 1:** Summary of algorithms used for signal detection.

Algorithms	Equation	Criteria
ROR	ROR = ad/bc 95% CI = e^ln(ROR) ± 1.96(1/a+1/b+1/c+1/d)^0.5^	lower limit of 95% CI > 1, a ≥ 3
PRR	PRR = a(c + d)/c(a + b) χ^2^= [(ad–bc)^2^](a + b + c + d)/[(a + b)(c + d)(a + c)(b + d)]	PRR ≥ 2, χ^2^ ≥ 4, a ≥ 3

a, number of reports containing both the suspect drug and the suspect adverse event; b, number of reports containing the suspect adverse event with other medications (except the drug of interest); c, number of reports containing the suspect drug with other adverse drug reactions (except the event of interest); d, number of reports containing other drugs and other adverse events. ROR, reporting odds ratio; PRR, proportional reporting ratio; CI, confidence interval; χ^2^, chi-squared.

## Results

### Descriptive analysis

**Fig. 1:**
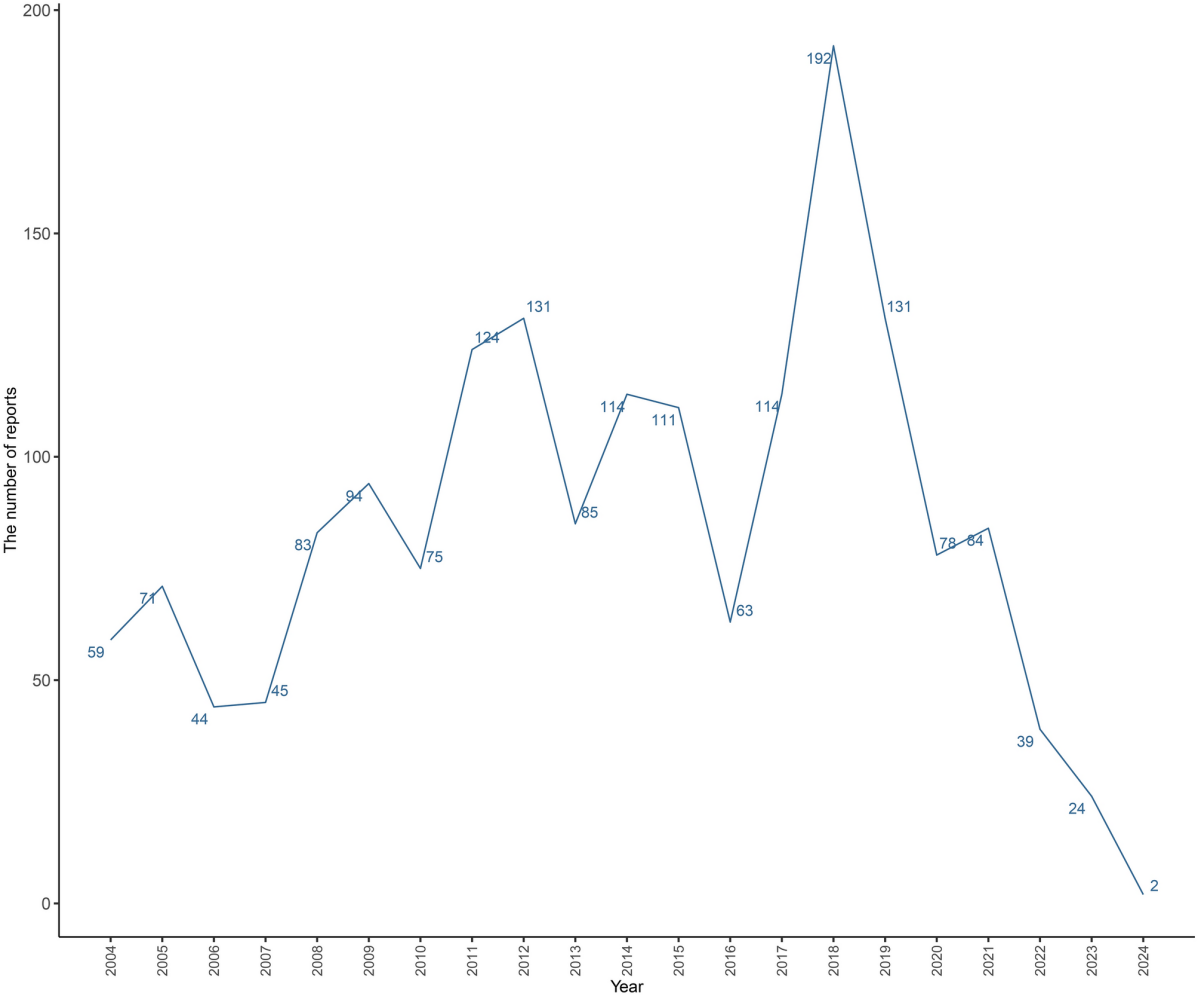
Number of reported cases from Q1 2004 to Q1 2024.

From Q1 2004 to Q1 2024, there were 20,754,281 AEs in FAERS database, of which 1763 were reported for anomaly of external ear congenital. As depicted in Fig. 1, the incidence of reported drug-induced anomaly of external ear congenital reached its peak in 2018, with 192 cases. Since 2007, the number of reports has risen in waves, with the largest increase of 81% from 2016 to 2017. The clinical characteristics of the patients are presented in Table 2. The majority of the affected individuals were male (477 cases, 42.32%), while females accounted for 34.69% (391 cases) of the reported cases. The age distribution revealed that the highest proportion of cases occurred in infants under the age of 1 (1589 cases, 90.13%), followed by those aged 10 years and above (43 cases, 3.82%). The median weight of the affected individuals was 2.75 kg (interquartile range: 2.17–3.40 kg). The most commonly reported outcomes associated with drug-induced anomaly of external ear congenital were congenital anomaly (47.72%), followed by hospitalization (10.30%), death (6.84%), disability (3.18%), life threatening (2.28%), and required intervention to Prevent Permanent Impairment/Damage (0.09%). Notably, the six most frequently reported clinical outcomes were all categorized as serious adverse events, which can substantially diminish patients’ quality of life. The median time to onset was 273 days, with an interquartile range of 137–311 days. In our study, we defined "time to onset" as the time from drug intake to the occurrence of the adverse event. This relatively long time to onset may be attributed to the fact that congenital malformations, such as external ear malformations, typically develop during the first trimester of pregnancy. The wide interquartile range observed in our study may be due to variations in the timing of drug exposure and individual differences in fetal development.

**Table 2:** Clinical characteristics of reported drug-induced anomaly of external ear congenital.

Variable	Total
*Sex*	
Female	391 (34.69)
Male	477 (42.32)
Unknown	895 (22.99)
*Age*	
< 1	1589 (90.13)
1–4	9 (0.80)
4–10	13 (1.15)
> = 10	43 (3.82)
Unknown	109 (6.18)
Weight	2.75 (2.17, 3.40)
*Reporter*	
Other health-professional	422 (37.44)
Physician	329 (29.19)
Consumer	144 (12.78)
Unknown	111 (9.85)
Pharmacist	86 (7.63)
Lawyer	35 (3.11)
*Reported countries*	
Other	633 (55.94)
United States	174 (15.44)
Germany	129(11.45)
France	86 (7.63)
United Kingdom	55 (4.88)
Denmark	50 (4.66)
*Outcomes*	
Congenital anomaly	1005 (47.72)
Other serious	623 (29.58)
Hospitalization	217 (10.30)
Death	144 (6.84)
Disability	67 (3.18)
Life threatening	48 (2.28)
Required intervention to prevent *Permanent impairment/damage*	2 (0.09)
Time to onset	273.00 (137.00, 311.00)

Among the countries with a significant number of reported cases, the United States had the highest number of reports (174 cases, 15.44%), followed by Germany (129 cases, 11.45%), France (86 cases, 7.63%), the United Kingdom (55 cases, 4.88%), and Denmark (50 cases, 4.66%). Based on the frequencies of AE reports, the top 50 drugs associated with congenital anomalies of the external ear. were summarized in Table 3. Valproic acid (122 cases) is the most frequently reported drug, followed by mycophenolate mofetil (105 cases), lamotrigine (65 cases), methotrexate (49 cases), accutane (37 cases), levetiracetam (29 cases).

**Table 3:** Top 50 drugs for signal strength.

Generic name (brand name)	Case reports	ROR	95% CI	PRR	95% CI	*X*^2^
Primidone (Mysoline)	4	397.05	147.21, 1070.9	388.71	145.89, 1035.7	1541.44
Valproic acid (Depakene)	122	239.46	123.75, 463.37	236.42	123.82, 451.43	2093.08
Methimazole (Tapazole)	3	198.35	63.49, 619.67	196.25	62.97, 611.67	581.23
Nevirapine (Viramune)	15	138.24	82.9, 230.51	137.23	82.44, 228.44	2001.63
Telbivudine (Sebivo)	5	117.1	48.51, 282.67	116.37	48.17, 281.12	569.39
Abacavir sulfate + lamivudine (Epzicom)	4	107.18	40.04, 286.89	106.57	40, 283.95	416.85
Allopurinol (Zyloprim)	17	84.25	49.68, 142.89	83.88	49.41, 142.39	1132.28
Isotretinoin (Accutane)	37	63.06	23.59, 168.6	62.85	23.59, 167.46	242.62
Carbamazepine (Tegretol)	21	57.6	35.13, 94.42	57.42	35.18, 93.73	874.49
Mycophenolate mofetil(CellCept)	105	55.48	42.09, 73.13	55.33	42.05, 72.8	2694.59
Lamotrigine (Lamictal)	66	50.69	36.02, 71.35	50.56	36.23, 70.55	1602.01
Misoprostol (Cytotec)	4	49.56	18.54, 132.46	49.43	18.55, 131.7	189.14
Ondansetron (Zofran)	15	43.84	26.32, 73.01	43.74	26.28, 72.81	618.1
Ritonavir (Norvir)	10	42.88	22.99, 79.97	42.78	22.85, 80.1	404.46
Lamivudine + zidovudine (Combivir)	6	37.12	16.63, 82.87	37.05	16.59, 82.75	209.35
Lopinavir + ritonavir (Kaletra)	14	35.32	20.84, 59.86	35.25	20.76, 59.84	460.16
Fluconazole (Diflucan)	6	32.32	14.48, 72.15	32.27	14.45, 72.08	180.82
Nevirapine (Viramune)	6	32.1	14.38, 71.65	32.04	14.34, 71.56	179.49
Abacavir + lamivudine (Kivexa)	3	30.83	9.92, 95.82	30.78	9.88, 95.93	86.2
Topiramate (Topamax)	20	30.52	16.37, 56.91	30.47	16.27, 57.05	282.54
Mycophenolic acid (Myfortic)	5	26.99	11.2, 65.01	26.95	11.16, 65.1	124.39
Paroxetine (Paxil)	19	24.17	9.05, 64.55	24.14	9.06, 64.32	88.42
Fluoxetine (Prozac)	25	23.69	12.71, 44.17	23.66	12.64, 44.3	215.1
Lamivudine (Epivir)	7	23.25	11.06, 48.91	23.23	11.03, 48.92	147.97
Methotrexate(Trexall)	49	22.51	16.54, 30.65	22.49	16.44, 30.77	830.34
Emtricitabine + tenof ovir disoproxil fumarate (Truvada)	4	21.87	8.19, 58.39	21.84	8.2, 58.19	79.27
Levocetirizine (Xyzal)	3	19.02	6.12, 59.1	19	6.1, 59.22	51.03
Naproxen (Aleve)	7	18.35	6.87, 49.01	18.34	6.88, 48.87	65.33
Levetiracetam (Keppra)	29	17.78	11.55, 27.39	17.77	11.55, 27.35	326.15
Dolutegravir (Tivicay)	3	16.79	5.4, 52.16	16.77	5.38, 52.27	44.38
Quetiapine (Seroquel)	12	15.5	7.73, 31.07	15.48	7.8, 30.74	107.63
Lacosamide (Vimpat)	3	15.41	4.96, 47.88	15.4	4.94, 48	40.29
Venlafaxine (Effexor)	12	15.06	6.75, 33.6	15.04	6.73, 33.59	78.25
Tacrolimus (Prograf)	12	12.02	6.8, 21.24	12.01	6.8, 21.2	119.88
Losartan(Cozaar)	3	11.42	3.68, 35.47	11.41	3.66, 35.56	28.43
Raltegravir (Isentress)	4	11.36	4.26, 30.33	11.36	4.26, 30.27	37.64
Imatinib (Gleevec)	3	10.54	3.39, 32.75	10.54	3.38, 32.85	25.83
Citalopram (Celexa)	8	10.19	5.08, 20.42	10.18	5.13, 20.22	65.77
Ramipril (Altace)	3	9.86	3.17, 30.63	9.86	3.16, 30.73	23.81
Diazepam (Valium)	3	9.55	3.07, 29.65	9.54	3.06, 29.73	22.88
Hydroxychloroquine (Plaquenil )	5	9.09	3.77, 21.88	9.08	3.76, 21.93	35.8
Escitalopram (Lexapro)	3	8.75	2.82, 27.17	8.74	2.8, 27.24	20.52
Letrozole (Femara)	3	8.46	2.72, 26.26	8.45	2.71, 26.34	19.66
Lisinopril (Prinivil)	4	7.66	2.87, 20.45	7.66	2.87, 20.41	23.07
Tacrolimus (Prograf)	4	5.19	1.95, 13.86	5.19	1.95, 13.83	13.49
Prednisone (Deltasone)	3	5	1.61, 15.54	5	1.6, 15.58	9.59
Cyclophosphamide (Cytoxan)	3	4.94	1.59, 15.35	4.94	1.58, 15.4	9.4
Gabapentin (Neurontin)	6	3.56	1.6, 7.94	3.56	1.59, 7.95	10.98
Metformin (Glucophage)	5	2.58	1.07, 6.2	2.58	1.07, 6.23	4.8

### Disproportionality analysis

The top 50 drugs with the highest signal strength are listed in Table 3. The drug with the highest ROR for congenital external ear malformations is primidone, with an ROR of 397.05 (95% CI 147.21, 1070.9). This is followed by valproic acid (ROR: 239.46, 95% CI 123.75, 463.37), methimazole (ROR: 198.35, 95% CI 63.49, 619.67), nevirapine (ROR: 138.24, 95% CI 82.9, 230.51), and telbivudine (ROR: 117.1, 95% CI 48.51, 282.67). Other notable drugs in the top 10 include abacavir sulfate + lamivudine (ROR: 107.18, 95% CI 40.04, 286.89), allopurinol (ROR: 84.25, 95% CI 49.68, 142.89), isotretinoin (ROR: 63.06, 95% CI 23.59, 168.6), and carbamazepine (ROR: 57.6, 95% CI 35.13, 94.42). Based on the criteria for PRR, the top 50 drugs with the highest PRRs were consistent with the results of RORs, The drug with the highest PRR for congenital external ear malformations is primidone, with a PRR of 388.71 (95% CI 145.89, 1035.7; chi-square: 1541.44). This is followed by valproic acid (PRR: 236.42, 95% CI 123.82, 451.43; chi-square: 2093.08), methimazole (PRR: 196.25, 95% CI 62.97, 611.67; chi-square: 581.23), nevirapine (PRR: 137.23, 95% CI 82.44, 228.44; chi-square: 2001.63), and telbivudine (PRR: 116.37, 95% CI 48.17, 281.12; chi-square: 569.39). Other notable drugs in the top 10 include abacavir sulfate + lamivudine (PRR: 106.57, 95% CI 40, 283.95; chi-square: 416.85), methimazole (PRR: 90.72, 95% CI 29.11, 282.75; chi-square: 265.51), allopurinol (PRR: 83.88, 95% CI 49.41, 142.39; chi-square: 1132.28), isotretinoin (PRR: 62.85, 95% CI 23.59, 167.46; chi-square: 242.62), and carbamazepine (PRR: 57.42, 95% CI 35.18, 93.73; chi-square: 874.49).

## Discussion

Congenital anomalies of the external ear can have a profound impact on a child’s physical, psychological, and social well-being. These malformations can lead to hearing impairment, difficulties with speech and language development, and social stigmatization. Children with external ear anomalies often require complex and multidisciplinary care, including surgical interventions, hearing rehabilitation, and ongoing support to address the challenges they face in their daily lives.

Several studies have previously reported associations between maternal drug exposure and an increased risk of congenital anomalies of the external ear. One of the most well-known examples is the thalidomide tragedy of the 1960s. Thalidomide, a sedative and anti-nausea medication, was found to cause severe birth defects, including ear malformations, when taken during early pregnancy^9^. This discovery led to increased awareness of the potential teratogenic effects of drugs and prompted stricter regulations for drug testing and approval. In more recent years, various studies have investigated the link between specific drugs and congenital external ear anomalies. A study by Anderka et al. found an association between maternal use of vasoactive drugs, such as pseudoephedrine and phenylpropanolamine, during early pregnancy and an increased risk of microtia, a congenital malformation characterized by underdevelopment of the external ear^6^. Another study by Alwan et al. examined the relationship between maternal use of nonsteroidal anti-inflammatory drugs (NSAIDs) and the risk of congenital anomalies. The results suggested a potential association between NSAID use during early pregnancy and an increased risk of microtia^11^ A systematic review investigated the association between maternal exposure to retinoic acids, such as isotretinoin, and the risk of congenital malformations. The review found that maternal use of retinoic acids was associated with an increased risk of craniofacial anomalies, including microtia and anotia (absence of the external ear). In addition to these studies, several case reports have documented instances of congenital external ear anomalies following maternal exposure to various drugs. For example, a case report described a case of bilateral microtia in an infant whose mother had taken the antiepileptic drug carbamazepine during pregnancy^20^.

We performed a comprehensive analysis of the FAERS database to investigate the adverse events, summarizing their clinical characteristics and mining the drugs that were most strongly associated with the development of these malformations. Based on the frequencies of AE reports and disproportionality analysis, we identified the top drugs with the highest signal strength for these malformations. The ROR and PRR were calculated to generate hypotheses on possible associations between drugs and the adverse events. To our knowledge, this research is the first and largest study conducted to assess the adverse events of drug-induced congenital anomalies of the external ear in real-world data based on analyzing the FAERS pharmacovigilance database. We described the clinical characteristics of these AEs and mined the drugs that were most strongly associated with congenital external ear anomalies. According to the disproportionality analysis, the top drugs with the highest ROR and PRR for congenital external ear anomalies were primidone, valproic acid, methimazole, nevirapine, and telbivudine.

Our findings are consistent with previous research linking valproic acid, an antiepileptic drug, to a range of congenital malformations, including craniofacial anomalies. Valproic acid, a widely used antiepileptic drug, has been well-documented to cause a range of congenital malformations, including neural tube defects, craniofacial anomalies, and limb defects^21^. Our study further supports the teratogenic potential of valproic acid, specifically in the context of external ear malformations. These findings are in line with the results of Rezaallah et al.^22^, who found that valproic acid may increase the risk of cleft lip and/or palate, another type of craniofacial malformation. While our study focused on external ear malformations and Rezaallah et al. primarily investigated cleft lip and/or palate, both studies contribute to a more comprehensive understanding of the reproductive risks associated with antiepileptic drugs, particularly valproic acid. However, there are several notable differences between our study and the research conducted by Rezaallah et al. First, our research specifically focused on the association between valproic acid and external ear malformations, while Rezaallah et al. primarily investigated the risk of cleft lip and/or palate associated with various antiepileptic drugs, including valproic acid. This difference in focus highlights the potential for valproic acid to cause a spectrum of craniofacial malformations. Second, our study utilized disproportionality analysis, whereas Rezaallah et al. reviewed Drug Analysis Prints from the UK Medicines and Healthcare products Regulatory Agency. Additionally, they performed descriptive analyses of relevant Summary of Product Characteristics and Patient Information in the UK and the USA. Third, while our study specifically focused on valproic acid, Rezaallah et al. investigated a broader range of antiepileptic drugs, including carbamazepine, lamotrigine, levetiracetam, and topiramate, among others. This broader scope allowed them to compare the teratogenic potential of various antiepileptic drugs. Fourth, our study provided a clear recommendation for healthcare professionals to carefully consider the risks and benefits of valproic acid use in women of childbearing age and to switch to alternative medications with a lower teratogenic potential when necessary. In contrast, Rezaallah et al. called for providing comprehensive and objective information to doctors and women of childbearing age, without offering specific recommendations for clinical decision-making. Finally, while our study did not have a specific geographical focus, Rezaallah et al. compared the safety information provided in SmPCs and PIs between the UK and the USA, highlighting differences in the comprehensiveness of the information provided.

Despite these differences, both studies contribute to a growing body of evidence on the teratogenic potential of antiepileptic drugs, particularly valproic acid, and emphasize the importance of informed decision-making when considering the use of these medications in women of childbearing age. Healthcare professionals should carefully consider the risks and benefits of valproic acid use in women of childbearing age and, if necessary, switch to alternative medications with a lower teratogenic potential. Combining the findings of both studies can inform clinical decision-making and help manage the risks associated with antiepileptic drug use during pregnancy.

Similarly, the association between nevirapine, an antiretroviral drug, and an increased risk of congenital anomalies, including ear malformations, has been reported in a previous study by Knapp et al.^23^. However, the package insert for nevirapine does not explicitly mention the risk of external ear malformations. Given the findings of our study and previous research, it may be necessary to update the package insert to include this specific risk, allowing healthcare professionals to make more informed decisions when prescribing nevirapine to pregnant women or women planning to become pregnant. Although not among the top five drugs, mycophenolate mofetil was the second (105 cases) most frequently reported drug associated with congenital anomalies of the external ear in our study. This finding is consistent with previous studies reporting an increased risk of congenital malformations, including ear anomalies, in infants exposed to MMF during pregnancy^24^.

Isotretinoin, indicated for severe acne treatment, demonstrated a strong signal for congenital external ear anomalies in our analysis (ROR 63.06, 95% CI 23.59–168.6; PRR 62.85). The teratogenic effects of isotretinoin are well-known, and the drug is contraindicated during pregnancy due to the high risk of congenital malformations, including craniofacial anomalies^25^. Our findings further support the need for strict pregnancy prevention measures and careful consideration of the risks and benefits when prescribing isotretinoin to women of childbearing age.

Notably, our disproportionality analysis revealed strong signals for congenital external ear malformations associated with primidone, methimazole, and telbivudine. These findings are of particular interest as the risk of congenital external ear malformations is not currently indicated in the package inserts for these medications. Primidone, an antiepileptic drug, had the highest ROR and PRR in our study, suggesting a strong association with congenital external ear malformations. The potential mechanism behind this association may be related to the drug’s effect on the development of the first and second branchial arches, which give rise to the external ear structures^1^. Additionally, primidone’s metabolite, phenobarbital, has been reported to cause congenital malformations in animal studies^26^, further supporting the teratogenic potential of primidone.

These findings highlight the need for further research to confirm the teratogenic potential of these drugs. The identification of these new associations may be attributed to the comprehensive nature of our study, which analyzed a large, real-world dataset from the FAERS database. The FAERS database contains spontaneous reports of adverse events submitted by healthcare professionals, patients, and pharmaceutical companies, allowing for the detection of rare or previously unknown drug-related adverse events^12^.

Healthcare professionals should be aware of the potential teratogenic effects of the drugs identified in this study and carefully consider the risks and benefits when prescribing them to women of childbearing age. In cases where the use of these drugs is necessary, patients should be informed of the potential risks and closely monitored throughout their pregnancy. Additionally, healthcare professionals should report any suspected drug-related adverse events, including congenital anomalies, to the appropriate regulatory agencies to contribute to the growing body of safety data and help inform clinical decision-making. Further research is needed to elucidate the mechanisms underlying the associations between these drugs and congenital external ear malformations. In vitro and animal studies may help to identify the specific developmental pathways and molecular targets affected by these drugs, providing insights into the pathogenesis of drug-induced congenital anomalies. Additionally, prospective studies with larger sample sizes and more detailed exposure data could help confirm these findings and provide more robust evidence for clinical decision-making. Such studies could also investigate the role of potential confounding factors, such as maternal age, comorbidities, and concomitant medication use, in the development of congenital external ear malformations.

One of the primary strengths of our study is the use of the FAERS database, which provides a large, real-world dataset for investigating potential associations between drug exposure and congenital anomalies of the external ear. The database includes reports from a diverse population, allowing for a more comprehensive analysis compared to smaller, single-center studies. Additionally, our use of disproportionality analysis helps to minimize the impact of confounding factors and reporting biases.

However, we acknowledge several limitations in our study. Firstly, the FAERS database relies on voluntary reporting, which may not be representative of the general population. Underreporting or overreporting of adverse events can introduce potential selection biases. To mitigate these biases, we focused on severe adverse events and direct reports submitted by consumers or healthcare professionals. Nevertheless, we recognize that these measures may not entirely eliminate the impact of reporting biases on our findings. Secondly, the lack of detailed genetic data in the FAERS database is a significant limitation of our study. Genetic factors play a crucial role in the development of congenital anomalies, and without this information, we cannot conclusively determine the extent to which genetic factors may have influenced the observed associations between drug exposure and external ear anomalies. Future studies incorporating genetic data would be essential to better understand the interplay between genetic predisposition and environmental factors, such as drug exposure, in the etiology of these anomalies. Lastly, as with any observational study, our findings demonstrate associations but do not establish causality. Confounding factors may influence the observed relationships between drug exposure and congenital anomalies. While we have taken steps to minimize the impact of confounding factors, such as focusing on primary suspect drugs and excluding secondary suspect drugs, residual confounding may still be present.

## Conclusion

In conclusion, this study identified several drugs associated with an increased risk of congenital external ear malformations using data from the FAERS database. While some of these drugs have package inserts that mention the risk of congenital malformations, others do not specifically address external ear malformations. These findings underscore the importance of continuous pharmacovigilance and the need for healthcare professionals to stay informed about potential drug-induced adverse events. A more comprehensive approach, involving multiple lines of evidence, is necessary to make informed decisions regarding drug safety and labeling.

## Data Availability

The data used during the current study are available from FAERS database (https://open.fda.gov/data/faers/).

## References

[1] Luquetti, D. V. *et al.* Microtia: Epidemiology and genetics. *Am. J. Med. Genet. A* **158A**(1), 124–139 (2012). 22106030 10.1002/ajmg.a.34352 PMC3482263

[2] Canfield, M. A. *et al.* Epidemiologic features and clinical subgroups of anotia/microtia in Texas. *Birth Defects Res. Part A, Clin. Mole. Teratol.* **85**(11), 905–913 (2009). 10.1002/bdra.20626 19760683

[3] Alasti, F. & Van Camp, G. Genetics of microtia and associated syndromes. *J. Med. Genet.* **46**(6), 361–369 (2009). 19293168 10.1136/jmg.2008.062158

[4] Harris, J., Källén, B. & Robert, E. The epidemiology of anotia and microtia. *J. Med. Genet.* **33**(10), 809–813 (1996). 8933331 10.1136/jmg.33.10.809 PMC1050757

[5] Mastroiacovo, P. *et al.* Epidemiology and genetics of microtia-anotia: A registry based study on over one million births. *J. Med. Genet.* **32**(6), 453–457 (1995). 7666397 10.1136/jmg.32.6.453 PMC1050485

[6] Anderka, M. T. *et al.* Reviewing the evidence for mycophenolate mofetil as a new teratogen: Case report and review of the literature. *Am. J. Med. Genet. Part A* **149A**(6), 1241–1248 (2009). 19441125 10.1002/ajmg.a.32685

[7] Lammer, E. J. *et al.* Retinoic acid embryopathy. *N. Eng. J. Med.* **313**(14), 837–841 (1985). 10.1056/NEJM198510033131401 3162101

[8] Carey, J. C. *et al.* Determination of human teratogenicity by the astute clinician method: Review of illustrative agents and a proposal of guidelines. *Birth Defects Res. Part A, Clin. Mole. Teratol.* **85**(1), 63–68 (2009). 10.1002/bdra.20533 19107954

[9] Vargesson, N. Thalidomide-induced teratogenesis: History and mechanisms. *Birth Defects Res., Part C, Embryo Today: Rev.* **105**(2), 140–156 (2015). 10.1002/bdrc.21096 PMC4737249 26043938

[10] Anderka, M. *et al.* Medications used to treat nausea and vomiting of pregnancy and the risk of selected birth defects. *Birth Defects Res., Part A Clin. Mole. Teratol.* **94**(1), 22–30 (2012). 10.1002/bdra.22865 PMC3299087 22102545

[11] Alwan, S. *et al.* Use of selective serotonin-reuptake inhibitors in pregnancy and the risk of birth defects. *N. Engl. J. Med.* **356**(26), 2684–2692 (2007). 17596602 10.1056/NEJMoa066584

[12] Zhou, X. *et al.* Epidemiology of congenital malformations of the external ear in Hunan Province, China, from 2016 to 2020. *Medicine* **103**(15), e37691 (2024). 38608109 10.1097/MD.0000000000037691 PMC11018175

[13] Duggirala, H. J. *et al.* Use of data mining at the food and drug administration. *J. Am. Med. Inform. Assoc. JAMIA* **23**(2), 428–434 (2016). 26209436 10.1093/jamia/ocv063 PMC11740535

[14] Böhm, R. *et al.* OpenVigil–free eyeballs on AERS pharmacovigilance data. *Nat. Biotechnol.* **30**, 137–138 (2012). 22318027 10.1038/nbt.2113

[15] Tian, X. *et al.* Cardiac disorder-related adverse events for aryl hydrocarbon receptor agonists: A safety review. *Expert Opin. Drug Saf.* **21**(12), 1505–1510 (2022). 35582860 10.1080/14740338.2022.2078301

[16] Caster, O. *et al.* Disproportionality analysis for pharmacovigilance signal detection in small databases or subsets: Recommendations for limiting false-positive associations. *Drug Saf.* **43**(5), 479–487 (2020). 32008183 10.1007/s40264-020-00911-w PMC7165139

[17] Ooba, N. & Kubota, K. Selected control events and reporting odds ratio in signal detection methodology. *Pharmacoepidemiol. Drug Saf.* **19**(11), 1159–1165 (2010). 20669233 10.1002/pds.2014

[18] Evans, S. J., Waller, P. C. & Davis, S. Use of proportional reporting ratios (PRRs) for signal generation from spontaneous adverse drug reaction reports. *Pharmacoepidemiol. Drug Saf.* **10**(6), 483–486 (2001). 11828828 10.1002/pds.677

[19] Fusaroli, M. *et al.* The Reporting of a disproportionality analysis for Drug safety signal detection using individual case safety reports in PharmacoVigilance (READUS-PV): Explanation and elaboration. *Drug Saf.* **47**(6), 585–599 (2024). 38713347 10.1007/s40264-024-01423-7 PMC11116264

[20] Eros, E. *et al.* A population-based case-control teratologic study of nitrazepam, medazepam, tofisopam, alprazolum and clonazepam treatment during pregnancy. *Eur. J. Obstet., Gynecol. Reprod. Biol.* **101**(2), 147–154 (2002). 11858890 10.1016/s0301-2115(01)00545-0

[21] Hill, D. S. *et al.* Teratogenic effects of antiepileptic drugs. *Exp. Rev. Neurother.* **10**(6), 943–959 (2010). 10.1586/ern.10.57 PMC2970517 20518610

[22] Rezaallah, B. *et al.* Risk of cleft lip and/or palate associated with antiepileptic drugs: Postmarketing safety signal detection and evaluation of information presented to prescribers and patients. *Ther. Innov. Regul. Sci.* **53**(1), 110–119 (2019). 29714593 10.1177/2168479018761638

[23] Knapp, K. M. *et al.* Prevalence of congenital anomalies in infants with in utero exposure to antiretrovirals. *Pediatric Infect. Dis. J.* **31**(2), 164–170 (2012). 10.1097/INF.0b013e318235c7aa PMC3261302 21983213

[24] Hoeltzenbein, M. *et al.* Teratogenicity of mycophenolate confirmed in a prospective study of the European network of teratology information services. *Am. J. Med. Genet. Part A* **158A**(3), 588–596 (2012). 22319001 10.1002/ajmg.a.35223

[25] AltıntaşAykan, D. & Ergün, Y. Isotretinoin: Still the cause of anxiety for teratogenicity. *Dermatol Ther.* **33**(1), e13192 (2020). 31837244 10.1111/dth.13192

[26] Bittigau, P., Sifringer, M. & Ikonomidou, C. Antiepileptic drugs and apoptosis in the developing brain. *Ann New York Acad Sci* **993**(103–114), 123–124 (2003). 10.1111/j.1749-6632.2003.tb07517.x 12853301

